# More Than Meets the Eye: The Merging of Perceptual and Conceptual Knowledge in the Anterior Temporal Face Area

**DOI:** 10.3389/fnhum.2016.00189

**Published:** 2016-05-02

**Authors:** Jessica A. Collins, Jessica E. Koski, Ingrid R. Olson

**Affiliations:** ^1^Frontotemporal Dementia Unit, Department of Neurology, Massachusetts General Hospital and Harvard Medical SchoolCharlestown, MA, USA; ^2^Department of Psychology, University of Texas AustinAustin, TX, USA; ^3^Department of Psychology, Temple UniversityPhiladelphia, PA, USA

**Keywords:** face processing, anterior temporal lobes, semantic memory, perception, multivoxel pattern analysis

## Abstract

An emerging body of research has supported the existence of a small face sensitive region in the ventral anterior temporal lobe (ATL), referred to here as the “anterior temporal face area”. The contribution of this region in the greater face-processing network remains poorly understood. The goal of the present study was to test the relative sensitivity of this region to perceptual as well as conceptual information about people and objects. We contrasted the sensitivity of this region to that of two highly-studied face-sensitive regions, the fusiform face area (FFA) and the occipital face area (OFA), as well as a control region in early visual cortex (EVC). Our findings revealed that multivoxel activity patterns in the anterior temporal face area contain information about facial identity, as well as conceptual attributes such as one’s occupation. The sensitivity of this region to the conceptual attributes of people was greater than that of posterior face processing regions. In addition, the anterior temporal face area overlaps with voxels that contain information about the conceptual attributes of concrete objects, supporting a generalized role of the ventral ATLs in the identification and conceptual processing of multiple stimulus classes.

## Introduction

Over a decade of neuroimaging work has characterized the neural basis of face perception and identified several nodes that preferentially respond to faces relative to other objects (Haxby et al., 2000; Kanwisher and Yovel, 2006). Most of this work has focused on the fusiform face area (FFA) and the occipital face area (OFA; Kanwisher et al., 1997; Kanwisher and Yovel, 2006; Pitcher et al., 2007), however an emerging literature has implicated an anterior temporal face area, on the ventral surface of the right anterior temporal lobes (vATLs) in or near perirhinal cortex, in facial processing (Tsao et al., 2008; Pinsk et al., 2009; Rajimehr et al., 2009; Avidan et al., 2013).

The function of the anterior temporal face area remains unclear. One body of research implicates this region in perception. Using single-cell recordings it has been shown that the identity of previously unfamiliar faces is represented in the population code of monkey vATL face areas (Freiwald and Tsao, 2010). In humans, discrete portions of the vATLs have the ability to discriminate between individual unfamiliar faces, as reported by two fMRI studies using multivoxel pattern analysis (MVPA; Kriegeskorte et al., 2007; Nestor et al., 2011). More recently it was shown that identity representations in the anterior temporal face area are sensitive to perceptual manipulations that affect identity, but not to perceptual manipulations that leave identity intact such as changes in rotation or lighting (Nasr and Tootell, 2012; Anzellotti et al., 2014). Furthermore, it has recently been shown that image invariant identity representations persist in the right vATL face region of a patient with focal damage to the OFA and FFA (Yang et al., 2016).

However, other work has pointed to a mnemonic function. The vATLs have been consistently implicated in semantic dementia, and individuals with this disorder frequently experience difficulty remembering biographical information about specific people (Evans et al., 1995; Snowden et al., 2004). Similar deficits have been observed in patients with focal unilateral lesions (reviewed by Olson et al., 2007). Damasio et al. (1990) coined the term “amnesic associative prosopagnosia” for patients with unilateral anterior temporal lobe (ATL) resections, as these individuals typically can discriminate individual faces based on their perceptual features, but fail to recognize the faces of familiar individuals. More recently, numerous studies have reported that portions of the ATLs are preferentially sensitive to familiar relative to novel faces, presumably due to the wealth of semantic information that is retrieved upon viewing a familiar face (reviewed by Von Der Heide et al., 2013). These effects appear to be lateralized, with the left ATL playing a role in retrieving names (Tsukiura et al., 2002, 2010), and the right ATLs playing a role in the retrieval of person- specific semantic information, such as their occupation, from facial cues (Tsukiura et al., 2002, 2006, 2008). In the episodic memory literature, it has been shown that activity patterns in perirhinal cortex, overlapping with face-selective voxels, differentiate between unfamiliar and recently learned familiar faces that have no semantic content (Martin et al., 2013, 2015).

We have recently proposed that the anterior temporal face area may perform person identification by integrating abstracted perceptual information with person-specific semantic knowledge (Collins and Olson, 2014). Here we started to test this hypothesis by using MVPA to assess the sensitivity of the anterior temporal face area to different aspects of newly learned person information: identity, occupation, and the setting in which they are typically encountered. We compared the sensitivity profile of the anterior temporal face area to that of the more posterior face processing regions (the OFA and FFA) and to a control region in early visual cortex (EVC). In addition, we assessed whether the anterior temporal face area overlapped with voxels in the vATLs that represent semantic information about non-social items: common objects.

## Materials and Methods

### Data Acquisition

Neuroimaging sessions were conducted at the Temple University Hospital on a 3.0 T Siemens Verio scanner (Erlangen, Germany) using a 12-channel Siemens head coil. The functional runs were preceded by a high-resolution anatomical scan that lasted 9 min. The T1-weighted images were acquired using a three-dimensional magnetization prepared rapid acquisition gradient echo pulse sequence. Imaging parameters were as follows: 144 contiguous slices of 0.9766 mm thickness; repetition time (TR) = 1900 ms; echo time (TE) = 2.94 ms; FOV = 188 × 250 mm; inversion time = 900 ms; voxel size = 1 × 0.9766 × 0.9766; matrix size = 188 × 256; flip angle = 9°.

Visual stimuli were shown using a rear mounted projection system. The stimulus delivery was controlled by E-Prime Software (Psychology Software Tools Inc., Pittsburg, PA, USA) on a windows desktop located in the scanner control room. Responses were recorded using a four-button fiber optic response pad system.

Functional T2*-weighted images sensitive to blood oxygenation level-dependent contrasts were acquired using a gradient-echo echo-planar pulse sequence and automatic shimming. Imaging parameters were as follows: TR = 3 s; TE = 20 ms; FOV = 240 × 240; voxel size = 3 × 3 × 2.5 mm; matrix size = 80 × 80; flip angle = 90°, GRAPPA = 2. Thirty-eight interleaved slices with 2.5 mm thickness were acquired aligned to the AC-PC line, with full brain coverage. Data preprocessing and univariate analysis of fMRI data were performed using FEAT (fMRI Expert Analysis Tool) version 6.0, part of the software library of the Oxford Centre for Functional MRI of the Brain (fMRIB^1^). MVPA analysis was carried out using the Princeton MVPA Toolbox version 0.7.1 running on MATLAB R2012b, and with custom MATLAB Software.

Early imaging studies of face perception likely missed anterior activations because they used a restricted FOV that excluded the inferior temporal lobe from image acquisition, or because they suffered from the well known problem of imaging the ATLs: susceptibility artifacts and signal distortion due to the proximity of these regions to the nasal sinuses and ear canals (Devlin et al., 2000; Visser et al., 2010). We were thoughtful about this problem in designing our study and made several adjustments that optimized our signal to noise ratio. We used small slice-thickness (2.5 mm) which has been shown to reduce signal drop-out caused by variations in the static magnetic field within a voxel (Farzaneh et al., 1990; Olman et al., 2009; Carlin et al., 2012). We also used a short (TE, 20 ms), which has also been shown to reduce signal drop-out (Farzaneh et al., 1990; Olman et al., 2009).

To assess whether there was adequate sensitivity for signal detection in the ATLs, the temporal signal to noise ratio (tSNR) for each participant was calculated using the first run of the functional localizer, by dividing the mean of the time series by the residual error SD after pre-processing. Visual inspection of individual tSNR maps confirmed signal coverage in the ATLs of all subjects that was well within a proper sensitivity range (>40; Murphy et al., 2007). Some signal loss in the medial orbitofrontal cortex (e.g., gyrus rectus) was observed and varied between participants.

### Participants

Eighteen participants (8 females) were recruited from Temple University. All participants were between the ages of 18 and 30, without history of brain injury or psychiatric illness, had normal or corrected-to-normal vision, and were right-handed. Written informed consent was attained from each subject before the first training session, and participants were compensated monetarily for their time. One participant was excluded from all analyses due to excessive movement, and another was excluded due to a failure to complete the face-training paradigm, resulting in a final sample of 16 participants.

### Behavioral Training Sessions

Eight gray-scale images of real male faces, all lacking facial hair and glasses and facing forward, were used in the training paradigm (stimuli courtesy of Michael J. Tarr^2^). In addition, eight object images were selected from the Internet. Object stimuli consist of gray-scale images of eight different items that would typically be found in a kitchen or hospital (blood pressure pumps, thermometers, corkscrews, mixers), which could be sorted into four different object categories. All stimuli were 360 by 360 pixels and displayed on a white background.

**Figure 1:**
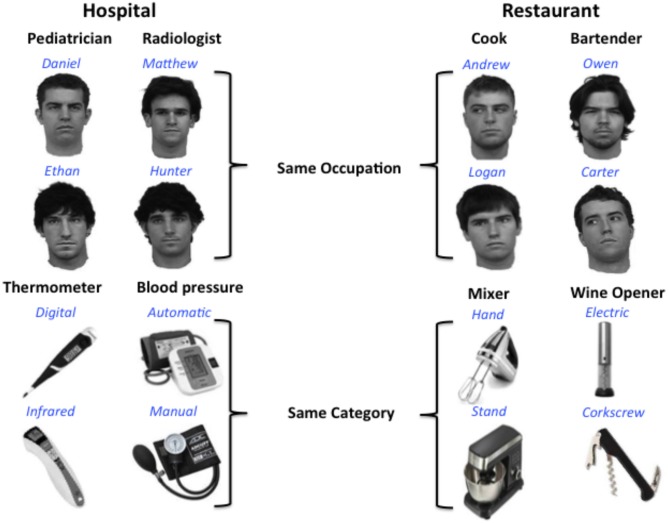
**Design for multivoxel pattern analysis (MVPA) analysis.** Participants learned to associate distinct faces and objects with identifying names (blue) and category labels or occupations (black). Each occupation or object category is typically found in one of two locations: a hospital or restaurant. Participants were not explicitly informed of the location associations.

Participants learned to associate a name and category label (occupation or object type) with each of the face and object stimuli (see Figure 1). Participants also learned to associate performance rankings (2 or 5 stars) with each face and object stimulus for a different project that will not be discussed here. To make object and face information as conceptually similar as possible, two object categories and two occupation categories were selected from two familiar public locations: restaurants and hospitals. Object categories with at least two different, distinct exemplars were selected, and these two exemplars represent the individual object names within each object category. Similarly, different male names were chosen to represent individual faces within each occupation category. Common, distinct male names were selected from the US Social Security list of the top 100 baby names for the 2000s.^3^ Training was conducted over 2 days in a laboratory setting, with the first session lasting approximately 45 min, and the second session lasting 30 min.

During the first training session, participants first completed “show” trials in which they passively viewed slides containing a face or object image, along with that face or object’s associated name and category labels. Each slide was presented four times each (64 trials total) for 5 s in a random order, and participants were instructed to learn the information for each face and object. Next participants completed “category response” trials, in which they viewed an image of a face or object, and were instructed to select the category that matched the displayed stimulus from a list of the four possible category labels presented below. The face and response choices remained on the screen until the participant responded, after which the correct category label was displayed for 2 s. Each face or object stimulus was presented twice (32 trials total) in a random order. Next participants completed 32 “status-response trials, ” during which each trained image was presented one at a time and participants selected the performance rating assigned to each item (either two or five stars). Following their response, the correct answer was displayed for 2 s. Participants completed blocks of trials in the following order, two times: show trials (64 total); category-response trials (32 total); show trials (64 total); and status-response trials (32 total).

The second training session used the same trial blocks in the same order as the first session. Afterwards, participants completed a free recall test, where a number was presented on the computer screen with one of the 16 trained images. Participants were instructed to write down on a separate sheet of paper all of the information they had learned for each image, including name, category, and status information.

Participants completed an additional recall test on the third day of the study, immediately before their fMRI session, to ensure they had retained all information learned during training. The recall test consisted of a piece of paper with the 16 trained images, and participants were instructed to write next to each face or object the associated name, category, and status information learned during training.

### Regions of Interest Task

A functional localizer scan preceded the experimental runs to localize areas sensitive to faces (the OFA, FFA, anterior temporal face area) and objects (the LOC, results reported elsewhere). The functional localizer utilized a block design, where seven types of images are presented: famous faces, non-famous faces, famous places, non-famous places, common objects, and scrambled images taken from publically available sources on the Internet. These stimuli were presented in gray-scale and varied in color, expression, pose, and background. The inclusion of famous faces in our functional localizer was motivated by our previous work (Von Der Heide et al., 2013) showing that that famous faces activate the same regions in the ATLs as non-famous faces, but that the activations tend to be larger and more robust. The images of famous faces and landmarks included here have been pilot tested to ensure that they are highly familiar within our study cohort (see Ross and Olson, 2012). An additional null stimulus was used, consisting of a gray screen and central fixation cross.

Images were randomly selected from lists of 89 images per category, and presented one at a time for 400 ms (350 ISI) in eight category blocks consisting of 14 trials. Participants performed a one-back task, responding whenever a randomly selected image was presented twice in a row (twice per block). The full cycle of category blocks was presented 5 times, with the order of categories varying in a fixed randomized order. Each cycle ended with a 12,000 ms presentation of a central fixation cross. The full localizer run lasted 9 min and 12 s and was completed twice.

### Regions of Interest Definition

The first five volumes of each run were discarded prior to any analyses. The following pre-processing steps were applied to all functional localizer data: non-brain removal using FSL’s brain extraction tool, motion correction using FSL’s MCFLIRT linear realignment tool, spatial smoothing using a 5 mm FWHM Gaussian kernel, high-pass temporal filtering with a 100 s cutoff, and un-distorting of the EPI data to correct for magnetic field distortions by means of individual field maps. EPI data were registered to each participant’s T1-weighted anatomical scan using BBR, and normalized to a standard Montreal Neurological institute (MNI-152) template.

After preprocessing the functional localizer runs for each fMRI time-series for each participant, the data were submitted to a fixed effects general linear model using FSL’s fMRI expert analysis tool (FEAT), with one predictor that was convolved with a double-gamma model of the hemodynamic response function (HRF) for each block type (face, places, fixation). Regions of interest (ROIs) were identified individuating the peaks showing greater activity for faces than for places (uncorrected, *p* < 0.05) in regions that have previously been implicated in face processing (for a review, see Collins and Olson, 2014). Spheres of 9 mm radius were generated, centered on the voxel with the highest activation within each peak. This ROI size (average = 106 voxels after intersection with the brain mask) was selected because it provided the best coverage of face-selective activations in the ATLs across individuals, and because it was consistent with previous MVPA studies that have demonstrated sensitivity to facial identity in the ATLs (Kriegeskorte et al., 2007; Goesaert and Op de Beeck, 2013; Anzellotti et al., 2014). Our face-selective ROIs included bilateral FFA located in the mid fusiform gyrus, the OFA, located in the inferior occipital gyrus, and the anterior temporal face area, located on the ventral surface of the ATLs. In some participants, multiple face-selective regions were identified in the ventral ATLs. For these participants the vATL functional ROI was centered on the most inferior peak, on the inferior temporal or fusiform gyrus, along the anterior collateral sulcus, consistent with previous work (Tsao et al., 2008; Rajimehr et al., 2009; Nestor et al., 2011; O’Neil et al., 2014). To control for effects driven by the low-level perceptual features of our stimuli, we additionally generated a 9-mm spherical ROI in EVC around the voxel showing the greatest activation for all visual stimuli (faces + places) vs. fixation within V1/BA17 as defined by the Juelich Histological Atlas in FSL. None of the face-selective ROIs overlapped in any participant at this sphere size, however the EVC and OFA ROIs overlapped partially in two participants. All ROIs were aligned to the individual subject’s (non-normalized) functional space using the FMRIB’s Linear Image Registration Tool. Given the wealth of data suggesting that the face-processing network is strongly lateralized to the right hemisphere (Sergent et al., 1992; Kanwisher et al., 1997; Rossion et al., 2003; Snowden et al., 2004; Gainotti, 2007, 2013, 2015; Gainotti and Marra, 2011; Duchaine and Yovel, 2015), we chose to restrict our analysis to face-processing ROIs (vATL, FFA, and OFA) in the right hemisphere, and bilateral EVC.

### MVPA Experimental Task and Design

Four novel exemplars of the same 8 male faces used during training were used in the experimental runs. These faces were angled 30° and 45° to the right or left. Four novel exemplars of each of the eight object types used during training were also used in the fMRI task. This is similar to prior work looking at object and face representations in the ATLs (see Peelen and Caramazza, 2012; Anzellotti et al., 2014) to avoid neural decoding of individual images, rather than object or facial identity.

The main experiment used a block design with a target detection task. The target detection task was orthogonal to the interests of the study in order to ensure that any activations observed were not due to task-related effects. Four exemplar images for each face and object identity (Fred, Carl, Hand-Mixer, Digital Thermometer, etc.,) from the training sessions were used for the main experiment. Stimuli were presented in 16 identity blocks (8 face, 8 object) per run, consisting of 16 trials each. Blocks were presented in a fixed random order, alternating between faces and objects. Within each block, each exemplar was presented 4 times in a fixed random order for 700 ms (425 ms ISI), and each block was followed by 3000 ms of fixation. Participants completed 16 blocks per run, lasting 5 min and 45 s each, and six total runs. Participants completed a target detection task in which they responded with a button press each time a green dot was displayed on an image. Target images appeared 3 times per block, with their exact locations following one of four predetermined patterns that varied across trial blocks in a fixed random order (to give the illusion that the order is completely random).

### MVPA Statistical Analysis

The first five volumes of each experimental run were discarded prior to any analyses. The following pre-processing steps were applied to all experimental data prior to our MVPA analysis: non-brain removal using FSL’s brain extraction tool, motion correction using FSL’s MCFLIRT linear realignment tool, high-pass temporal filtering with a 50 s cutoff, and un-distorting of the EPI data to correct for magnetic field distortions by means of individual field maps. EPI data were registered to each participant’s T1-weighted anatomical scan using BBR.

We used MVPA to assess the sensitivity of the functionally defined bilateral OFA, FFA, and anterior temporal face areas to two types of information associated with faces and objects: facial/object identity, and the category that the face or object belonged to. We additionally assessed the sensitivity of each of our functionally defined ROIs to the typical location of each object-type or person category (i.e., either a kitchen or hospital), regardless of stimulus type.

Across all analyses data were *z*-scored within each run to control for baseline shifts in the magnetic resonant signal, and all regressors were convolved with a standard HRF. For the first analysis we defined 8 regressors, one for each facial identity. We then assessed whether our classifier could identify unique multivoxel patterns for each facial identity. For the second analysis we defined four regressors, one for each occupation label, and assessed whether our classifier could identify unique multivoxel patterns for different faces that shared the same occupation. Next we performed analogous analyses assessing the ability of our classifiers to identify unique multivoxel patterns for distinct object identities, and for objects that shared a category label. Finally, we defined two regressors, one for each spatial location (Hospital vs. Kitchen) and assessed whether our classifier could identify unique multivoxel patterns for different faces and objects that are associated with the same location. We used a Gaussian Naïve Bayes (GNB) classifier and a leave-one-run-out cross validation scheme in which the classifier was trained on five runs of data and tested on the remaining un-trained run. This procedure was repeated 6 times, each time using a different test run, and the average classification accuracy was calculated for each ROI and compared to chance performance (face/object identity = 0.125, face/object category = 0.25, location = 0.5) using a one-tailed *t*-test. Following the methods of previous studies that have employed similar methods (Epstein and Morgan, 2012; Peelen and Caramazza, 2012; Goesaert and Op de Beeck, 2013; Anzellotti et al., 2014), all analyses were restricted to *a priori*-defined regions of interest and statistical significance is reported using an uncorrected threshold of *p* < 0.05. However in Figure 3 we will also indicate which ROIs have statistical effects that survive a conservative correction for multiple comparisons (*p* < 0.0125). For analyses in which multiple ROIs showed classification accuracy significantly above chance, differences in the classification accuracy of each ROI were compared using two-tailed *t*-tests to avoid making assumptions about the directionality of the effects.

## Results

### Behavioral Results

Participants were quite accurate at recollecting information about trained people and objects immediately following training (*M* = 0.98, *SD* = 0.05) and immediately prior to scanning (*M* = 0.97, *SD* = 0.04). Immediately after training recall performance for object category was significantly greater than for object name (*M* = 99% vs. 93%, *t*_(17)_ = 2.54, *p* = 0.021), with no other differences in recall accuracy being observed across any of the attributes tested. Likewise, participants were highly accurate at detecting targets across both functional localizer runs (*M* = 0.97, *SD* = 0.03) and all six experimental runs (*M* = 0.98, *SD* = 0.04) indicating attention to stimuli was maintained across the experiment.

### Functional Localizer

**Figure 2:**
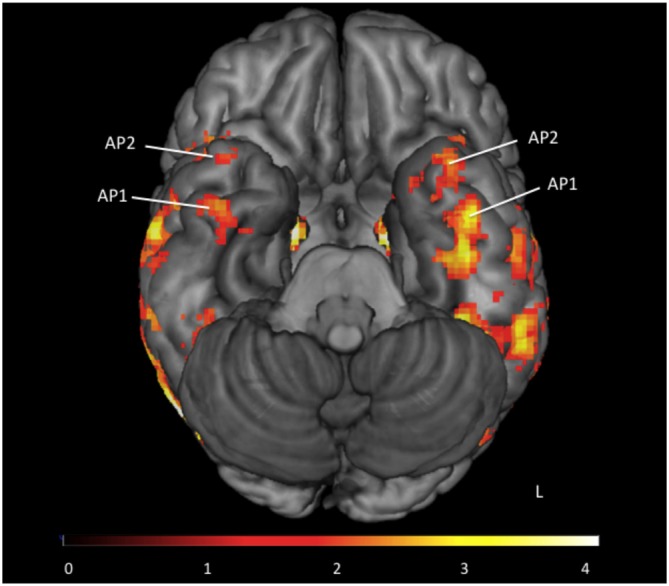
**Group map of anterior face temporal face areas.** Average activations for the contrast faces > places (thresholded *z* > 1.65) superimposed on a ventral view of the inferior surface of the brain. Anterior temporal faces areas 1 and 2 are labeled as first anterior temporal face area (AP1) and second anterior temporal face area (AP2) respectively. Color scale presented as *z*-scores.

All participants had 1 or 2 face-sensitive regions anterior to the FFA (see Figure 2). The first anterior temporal face area (AP1), was located on the inferior temporal or fusiform gyrus, along the anterior collateral sulcus. The second anterior temporal face area (AP2) was located more anteriorly, on the inferior or middle temporal gyrus near the temporal pole. These regions showed no laterality bias, and were found in locations consistent with the location of anterior temporal face-areas identified in earlier fMRI studies (Tsao et al., 2008; Rajimehr et al., 2009). Their location is also consistent with the facial identify area identified by Nestor et al. (2011). In the right hemisphere 13 subjects had both AP1 and AP2, and 3 subjects had AP1 only. In the left hemisphere 14 subjects had both AP1 and AP2, and 2 subjects had AP1 only. For consistency with previous literature (Rajimehr et al., 2009; Nasr and Tootell, 2012; Avidan et al., 2013; Axelrod and Yovel, 2013; Yang et al., 2016) our vATL ROI was located in right AP1 for all participants.

### Differential Discriminability in the Face-Processing Network

**Figure 3:**
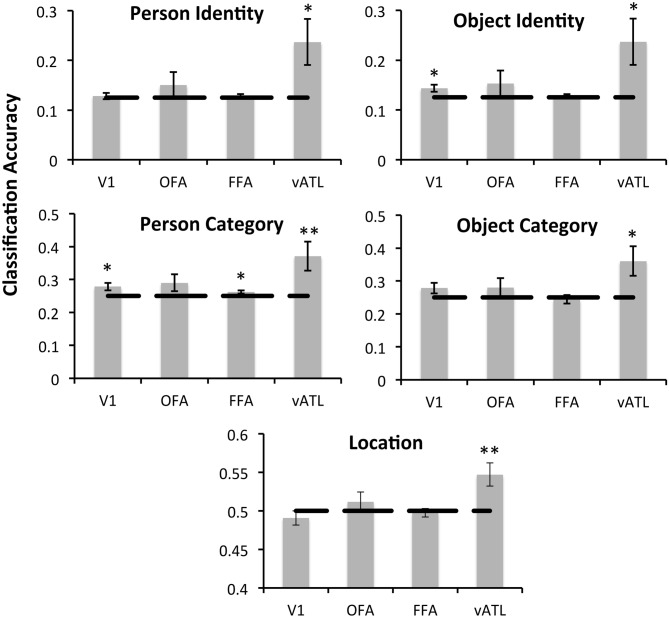
**The classification accuracy of each face-network regions of interest (ROI) for facial identity, facial occupation, object identity, object category, and location as assessed by the MVPA analysis.** The dashed line represents chance performance (face/object identity = 0.125, face/object category = 0.25, location = 0.5). Single asterisks (*) indicate significant above-chance classification accuracy at an uncorrected threshold as indicated by a one-tailed *t*-test (*p* < 0.05). Double asterisks (**) indicate above-chance classification accuracy that survives a Bonferroni correction for multiple comparisons (*p* < 0.0125). All face-selective ROIs were located in the right hemisphere.

To test for conceptual face representations in the face-processing network, we assessed the ability of each region to accurately classify facial identity and occupation (Figure 3). Facial identity was accurately decoded within the right anterior temporal face area [*t*_(15)_ = 2.21, *p* = 0.021], however this effect was not found in the right FFA, OFA or EVC (*p*’s > 0.1). Facial occupation was also accurately decoded by multivoxel patterns in the right anterior temporal face area [*t*_(15)_ = 2.52, *p* = 0.012] as well as the right FFA [*t*_(15)_ = 2.12, *p* = 0.026] and EVC [*t*_(15)_ = 2.27, *p* = 0.019]. The right OFA did not display sensitivity to facial occupation that was significantly above chance (*p* > 0.05) Classification accuracy for facial occupation was significantly higher in the right anterior temporal face area than in the FFA [*t*_(15)_ = 2.43, *p* = 0.028] and was marginally higher than in EVC [*t*_(15)_ = 1.94, *p* = 0.072].

Next we assessed the ability of each ROI to accurately classify different aspects of object information within its multivoxel activity pattern. Both the right anterior temporal face area [*t*_(15)_ = 2.20, *p* = 0.022] and EVC [*t*_(15)_ = 2.40, *p* = 0.015] were able to accurately classify objects according to their identity. Classification accuracy for object identity did not differ significantly in the anterior temporal face and EVC (*p* > 0.05). Classification accuracy for object category was also significantly above chance in the anterior temporal face area [*t*_(15)_ = 2.25, *p* = 0.020], but not in the, FFA, OFA, or EVC (*p*s > 0.05). Finally the ability of each face-processing region to accurately classify faces and objects according to where they are typically found (e.g., a doctor and stethoscope are typically found in a hospital) was assessed. Only the anterior temporal face area demonstrated sensitivity to conceptual knowledge about location that was significantly above chance, *t*_(15)_ = 2.89, *p* = 0.006. Exploratory follow-up analyses revealed no sensitivity to any of the contrasts of interest in any left-hemisphere ROI (vATL, FFA, or OFA, all *p’*s > 0.05).

## Discussion

The results reported here show that a face sensitive region in the ventral ATLs, dubbed the “anterior temporal face area”, represents information that goes beyond perceptual features of faces to include semantic information about a person’s identity and occupation. These findings are consistent with previous studies implicating the functionally-localized anterior temporal face area in the representation of facial identity (Anzellotti et al., 2014; Yang et al., 2016), and further demonstrate that this region may possess the unique ability to represent abstract conceptual information about an individual.

Interestingly, the FFA was sensitive to facial category, but not facial identity in our study. The existing literature is mixed in regards to this issue: four prior MVPA studies reported sensitivity to facial identity in the FFA (Nestor et al., 2011; Axelrod and Yovel, 2013, 2015; Goesaert and Op de Beeck, 2013; Anzellotti et al., 2014), while another MVPA study failed to find identity representations in the FFA (Kriegeskorte et al., 2007). To our knowledge only one study has demonstrated sensitivity in the FFA to semantic attributes associated with a person (van den Hurk et al., 2011).

### The vATLs: Face Memory vs. Face Perception

We have previously suggested that a region of the vATL plays a role in face perception as well as person memory (Collins and Olson, 2014). Here we extend these findings by showing that the functionally-defined anterior temporal face area is sensitive to the identity of a facial stimulus, as defined by its perceptual features across changes in viewpoint, as well as learned semantic attributes about individuals. Although the differential sensitivity of the vATL face region to different types of person information remains to be explored, based on the findings reported here, as well as a wealth of research demonstrating sensitivity of the ATLs generally to semantic knowledge (Collins and Olson, 2014), we speculate that the anterior temporal face area may be critically involved in the integration of perception and memory for the end goal of person identification. This proposal is consistent with previous cognitive models of face recognition that supported the existence of an amodal *person identity node* (PIN) that when activated, enabled the retrieval of person-specific semantic information (Bruce and Young, 1986). The anterior temporal face area is ideally suited to serve this function due to due to several special properties of this region.

First, the anterior temporal face area is sensitive to perceptual attributes of faces but in a highly restricted manner. For instance, we tested two humans with unilateral ATL resections across a range of face discrimination tasks using carefully controlled morphed face stimuli. We found that they performed normally on many difficult tasks requiring discrimination of facial gender or facial age, but performed abnormally low when required to discriminate facial identity defined by altering a gestalt representation (Olson et al., 2015). Our results, and those of Olson et al. (2015), mimic findings in macaques (Freiwald and Tsao, 2010) and humans (Freiwald and Tsao, 2010; Nasr and Tootell, 2012; Anzellotti et al., 2014) showing that cells in the vATLs are only sensitive to perceptual manipulations that alter facial identity, but are *insensitive* to many low-level perceptual manipulations that leave facial identity intact such as inversion, contrast reversal, and viewpoint. This region may even be insensitive to higher-level perceptual changes that leave identity intact such as changes in facial expression (Nestor et al., 2011).

Second, there is evidence from the episodic memory literature that portions of the ATLs are sensitive to a range of mnemonic manipulations: responsiveness is enhanced by knowledge-based familiarity in the form of semantic knowledge (Nieuwenhuis et al., 2012; Ross and Olson, 2012), but decreased by perceptual familiarity in the form of stimulus repetition (Sugiura et al., 2001, 2011). Cells in this region also have the ability to represent associative pairings (Brambati et al., 2010; Eifuku et al., 2010; Nieuwenhuis et al., 2012), and activity patterns in this region represent information about prior encounters with a facial image (Martin et al., 2013, 2015). One recent study showed that face-place associations were initially represented in the human hippocampus but after a 25 h delay were found to reside in the ATL (Nieuwenhuis et al., 2012). It has often been suggested that the hippocampus is responsible for the initial consolidation of associations but that a short time later, these representations are shipped out to various parts of the cortex, a notion supported by these findings. The tight structural interconnectivity of the vATLs, amygdala, and anterior hippocampus via short-range fiber pathways may facilitate this process (Insausti et al., 1987; Morán et al., 1987; Suzuki and Amaral, 1994; Blaizot et al., 2010).

### Object Sensitivity in the Anterior Temporal Face Area

Notably, our anterior temporal ROI contained voxels that also represented the identity and category of concrete objects, and implicit knowledge about where people or items are typically found (regardless of their stimulus domain). These findings are consistent with a rich neuropsychology literature showing reliable semantic memory deficits for concrete objects following cell loss in ventral aspects of the ATLs (Mion et al., 2010). Furthermore, these finding are also consistent with a recent MVPA study showing that activity patterns within the ventral ATLs represent the conceptual attributes of everyday objects, such as how they are used or where they are typically found (Peelen and Caramazza, 2012).

The sensitivity of the anterior temporal face area in both experiments to object knowledge may be driven by the inclusion of object-sensitive voxels in our ROIs. We chose to use the same ROI size for all face-selective regions as previous work has shown that the number of voxels within an ROI may influence MVPA outcomes (e.g., Eger et al., 2008; Anzellotti et al., 2014). Here, we selected to use a 9-mm sphere because it provided the best coverage of face-selective activations across individuals, and because it was consistent with previous MVPA studies that have demonstrated sensitivity to facial identity in the ATLs (Kriegeskorte et al., 2007; Goesaert and Op de Beeck, 2013; Anzellotti et al., 2014). Our 9 mm ROIs encompassed an average of 106 voxels in the right ATL after intersection with the brain mask, and extended beyond face-selective cortex in some individuals. Previous work has shown that the vATLs are sensitive to both to faces and objects (Barense et al., 2010, 2011; McLelland et al., 2014; Mundy et al., 2012), and further analysis of our own functional localizer data confirmed that spatially adjacent and overlapping voxels respond preferentially to faces and objects (See Supplementary Figure 1). An important avenue for future research will be to investigate, with high-resolution imaging techniques, the degree to which face- and object- selective voxels independently and jointly contribute to the representation of conceptual attributes about faces and objects. The sensitivity of the vATLs to object conceptual object properties does not render this area un-important for face processing; even the FFA shows some sensitivity to non-face objects (Gauthier et al., 1999, 2000; Grill-Spector et al., 2006; McGugin et al., 2012; Yang et al., 2016).

### Limitations

In designing our experiment we chose to use naturalistic stimuli that might reasonably be encountered in the real world, and thus applied only minimal stimulus editing by using gray-scale images. Thus, one possibility is that the sensitivity of the anterior temporal face area to the conceptual properties of faces is being driven by perceptual attributes of the stimuli; a contention that is supported by the sensitivity of the early visual ROI to facial category. We think that this is unlikely for several reasons, outlined here. First, all facial stimuli were arbitrarily paired to each occupation label, and we were careful not to group highly similar looking individuals into the same occupation category. Second, if our effects were being driven primarily by visual attributes of the facial stimuli with different occupation labels, we would expect to see higher classification accuracy for facial occupation in EVC than in the vATLs, which was not the case. Sensitivity to facial occupation was higher in the vATLs than in EVC, and did not survive correction for multiple comparisons in EVC. It is possible that that the sensitivity of the OFA to facial category is being driven by top-down feedback from higher-order areas (Bar et al., 2006), however future work is needed to investigate this possibility.

In order to ensure that the participants in our study adequately learned all stimulus associations we used a small stimulus set and a short delay between training and the fMRI session. Thus, participants may have recalled episodic details of the study context during the fMRI session in addition to the semantic attributes associated with each stimulus. Although the retrieval of episodic details may have contributed to the results reported here, we believe that the robust sensitivity of the anterior temporal face area to object and face identity and category, as well as to location, which was not explicitly reinforced during the training paradigm, precludes an expiation based solely on episodic memory retrieval. However, including a longer delay period between study and test, or using familiar faces that have a known association with an occupation or a location, would provide a stronger test of our hypothesis. Furthermore, additional work extending our paradigm to larger, more ecologically valid stimulus sets will lend further validity to our results.

## Conclusion

To conclude, the present study shows that conceptual knowledge about an individual’s identity and social category is represented in multivoxel activity patterns in the anterior temporal face area. Voxels in the anterior temporal face area also demonstrated sensitivity to conceptual knowledge associated with concrete objects, suggesting that it may play a role in representing conceptual knowledge about concrete objects more generally. Future research should investigate whether the same population of neurons is tuned to both faces and objects, or as we suspect, different populations. Our results are consistent with a recent model of face processing in which an organized system of face areas extends bilaterally from the inferior occipital gyri to the vATLs, with facial representations becoming increasingly complex and abstracted from low-level perceptual features as they move forward along this network (Collins and Olson, 2014). Our results further suggest that the anterior temporal face region may serve as an interface between face perception and face memory, linking perceptual representations of individual identity with person-specific semantic knowledge.

## Ethics Statement

The research was approved by the Temple University Institutional Review Board.

## Author Contributions

JAC was responsible for the original experimental design and fMRI data analysis. JEK collected the fMRI data and programed the experiments. JAC interpreted the results and wrote the article with input from JEK and IRO.

## Funding

This work was supported by a Temple University Dissertation Award to JAC and a National Institute of Health Grant to IRO (RO1 MH091113). The content is solely the responsibility of the authors and does not necessarily represent the official views of the National Institute of Mental Health or the National Institutes of Health.

## Conflict of Interest Statement

The authors declare that the research was conducted in the absence of any commercial or financial relationships that could be construed as a potential conflict of interest.

## Footnotes

^1^www.fmrib.ox.ac.uk/fsl

^2^www.tarrlab.org/

^3^www.socialsecurity.gov
