# Supplementary material for: More Than Meets the Eye: The Merging of Perceptual and Conceptual Knowledge in the Anterior Temporal Face Area
Source: Front Hum Neurosci. 2016 May 2;10:189. doi: 10.3389/fnhum.2016.00189 (PMC4852584; doi:10.3389/fnhum.2016.00189)
Supplement: Supplementary file 1 [file Image_1.pdf]

Supplementary Material

More Than Meets the Eye: The Merging of Perceptual and Conceptual Knowledge in the Anterior Temporal Face Area.

Jessica A. Collins*, Jessica E. Koski, Ingrid R. Olson

*** Correspondence:** Jessica A. Collins: JCollins21@mgh.harvard.edu

1 Supplementary Figures

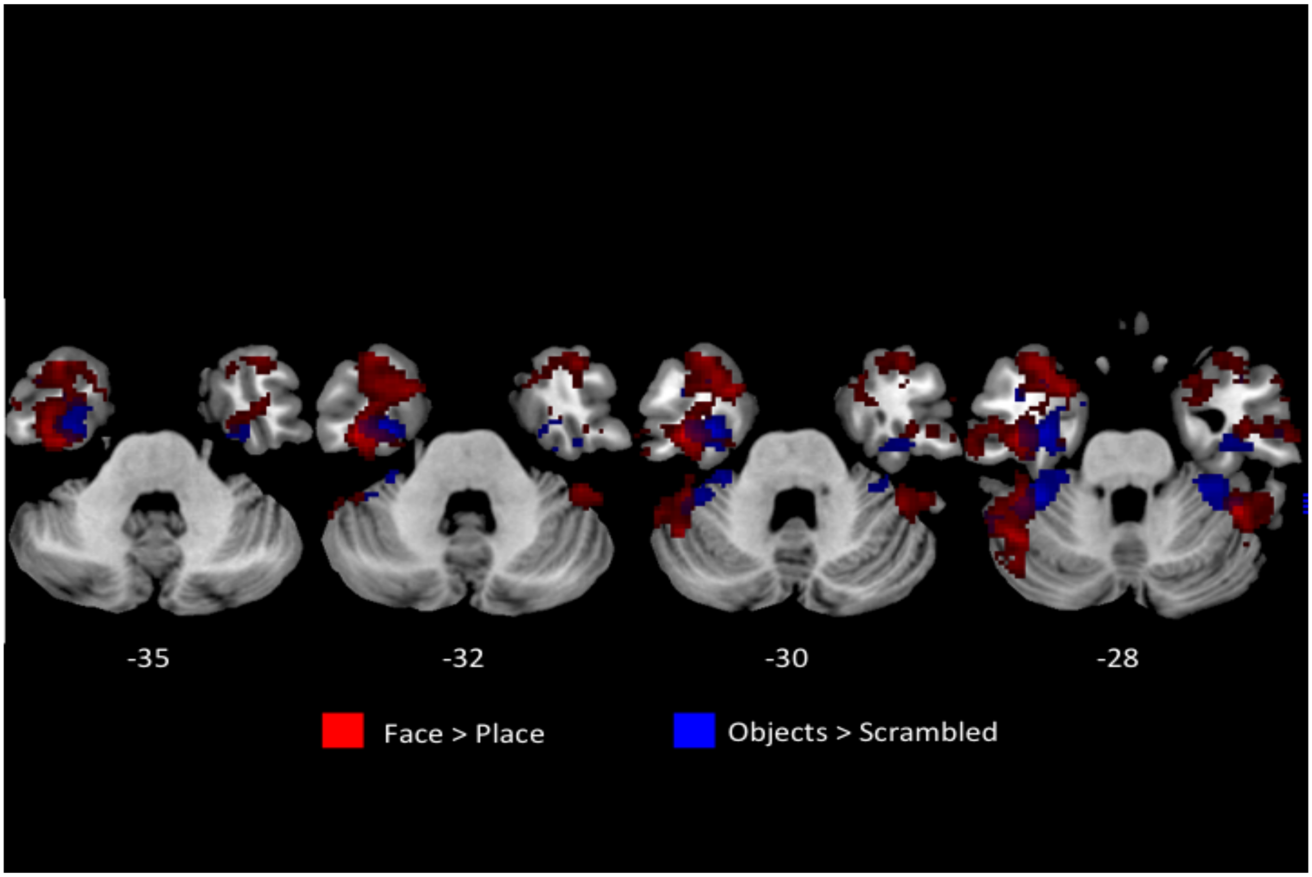

Supplementary Figure 1: Group Map of Face Selective and Object Selective Voxels. Average activations for the contrasts faces > places (z > 1) and objects > scrambled objects (z > 2) superimposed on axial slices. Numbers refer to the MNI y-coordinates.
